# Magnetotransport and ARPES studies of the topological insulators Sb_2_Te_3_ and Bi_2_Te_3_ grown by MOCVD on large-area Si substrates

**DOI:** 10.1038/s41598-022-07496-7

**Published:** 2022-03-10

**Authors:** L. Locatelli, A. Kumar, P. Tsipas, A. Dimoulas, E. Longo, R. Mantovan

**Affiliations:** 1 Institute for Microelectronics and Microsystems, CNR-IMM Unit of Agrate Brianza, Via C. Olivetti 2, 20864 Agrate Brianza, Italy; 2 Department of Material Science, University of Milano Bicocca, Via R. Cozzi 55, 20126 Milan, Italy; 3 National Centre for Scientific Research ‘DEMOKRITOS’, Patriarchou Grigoriou & Neapoleos 27, 15310 Agia Paraskevi, Athens, Greece

**Keywords:** Electronic devices, Information storage

## Abstract

Recently, the topological insulators (TIs) antimony telluride (Sb_2_Te_3_) and bismuth telluride (Bi_2_Te_3_) are attracting high interest for applications based on spin-charge interconversion mechanisms. Aiming to make a step toward the technology transfer, it is of major importance to achieve and investigate epitaxial quality-TIs on large area Si-based substrates. In view of that, we report here magnetotransport and angle-resolved photoemission spectroscopy (ARPES) studies on Sb_2_Te_3_ and Bi_2_Te_3_ thin films grown by metal organic chemical vapor deposition (MOCVD) on top of 4″ Si(111) substrates. Clear weak antilocalization (WAL) effects are observed in both TIs, proving the existence of quantum transport mechanism, and the data are successfully interpreted in the framework of the Hikami–Larkin–Nagaoka model. Further, by dedicated magnetotransport experiments, it has been confirmed that the investigated WAL originates from two-dimensional (2D) topological states. ARPES has been performed ex-situ, and in both TIs the gapless Dirac cones have been observed and attributed to the topological surface states. Combining the proofs of the existence of quantum 2D transport as deduced from the analysis of the magnetoconductance curve with the direct observation of the Dirac-like band structure revealed by the ARPES spectra, it is possible to unambiguously confirm the topological nature of our Sb_2_Te_3_ and Bi_2_Te_3_ thin films. The results obtained on thin films grown by MOCVD on 4’’ Si(111) substrate mark an important step towards the technology transfer of the topological insulators studied in this work.

## Introduction

The Bi_2_Se_3_, Bi_2_Te_3_, and Sb_2_Te_3_ chalcogenides are in the focus of interest as three-dimensional (3D) topological insulators (TIs), due to the well-documented existence of topologically-protected surface states (TSS)^1–4^. These conductive states are protected by the symmetry of the crystal, exhibiting a very high electronic mobility. The joint presence of TSS and the intrinsic high spin–orbit coupling (SOC) of 3D-TIs, makes them very promising candidates to boost the efficiency of spin-charge interconversion phenomena due to their higher efficiency in comparison to the workhorse heavy metals Pt or Ta^5–12^. Currently, the most widely employed method to grow TIs is molecular beam epitaxy (MBE)^13–15^ which could hardly sustain industrial needs. To facilitate the technology transfer of the 3D-TIs, the use of chemical methods is a very promising strategy^16,17^. Recently, we reported about the metal organic chemical vapor deposition (MOCVD) growth of epitaxial quality Sb_2_Te_3_ and Bi_2_Te_3_ on top of 4″ Si(111) substrates^18,19^, and the integration of Sb_2_Te_3_ with ferromagnetic thin films^20,21^, further demonstrating a high spin-to-charge (S2C) conversion^22,23^.

In this work, we combine temperature-dependent magnetotransport, and angle-resolved photoemission spectroscopy (ARPES) measurements, both performed on 1 × 1 cm^2^ samples, to undoubtfully demonstrate the topological character of the produced 3D-TIs over a macroscopic area.

The shape of a magnetoconductance (MC) curve embeds several information about the transport properties of a system. In particular, the existence of TSS in a topological material is revealed by the presence of a purely quantum mechanical phenomenon known as weak antilocalization (WAL), firstly studied by Hikami, Larkin and Nagaoka (HLN) in 1980^24^. The WAL effect is observed in systems where the spin of the carriers is conserved after various elastic scattering events in which only their momentum changes, in the so-called quantum diffusive regime^25^. Below a certain temperature, the WAL is observed in TIs due to the intrinsic high conductivity and helical polarization of their TSS^25,26^. However, it is important to highlight that the observation of WAL is a condition which is necessary, but not sufficient, to prove the existence of TSS. Indeed, this effect arises in good conductors where the electronic wave function maintains its phase coherence over a distance that is much larger than the electronic mean free path (MFP)^25^, and this is not a prerogative of TIs. Particularly, the two conditions needed to observe the WAL effect are verified also in bulk conductive materials whose lattice is composed by heavy elements, which by means of an intense Spin–Orbit Interaction (SOI) are responsible for the spin conservation over several elastic scattering events^25,26^. With the aim of understanding whether the observed WAL originates from a 2D transport mechanism, or from the bulk SOI, the MC curves were recorded at different angles between the surface of the film and the applied external magnetic field, demonstrating the 2D nature of the observed WAL. *Ex-situ* ARPES was employed to image the band structure of the films, revealing the typical Dirac-like dispersion relation^1,27–29^. By combining angular dependent magnetotransport and ARPES experiments, we conclude that our MOCVD-grown TIs host TSS, which actively participate to the transport mechanism. Thus, our work demonstrates the potential of MOCVD technique for fabricating functional TIs over large-area Si substrates, providing a substantial contribution to their possible practical uses.

## Results and discussion

### Transport and Hall effect measurements

**Figure 1:**
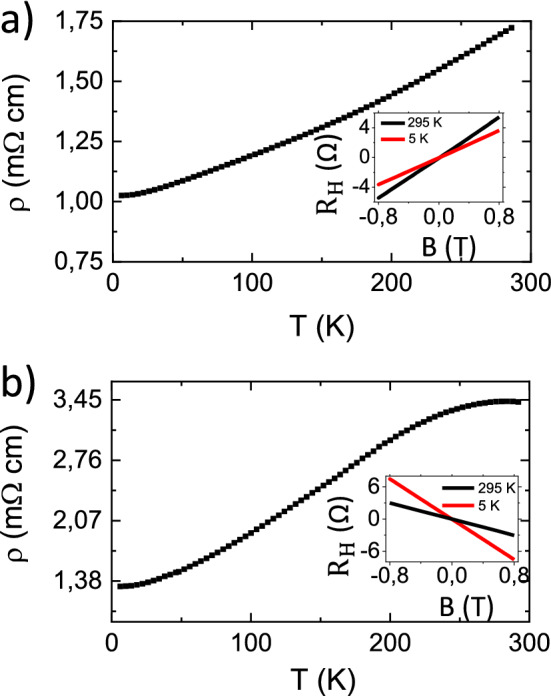
ρ as obtained for Sb_2_Te_3_ (**a**) and Bi_2_Te_3_ (**b**), in the Van der Pauw measurement geometry in the 5–295 K range. The insets in panels (**a**) and (**b**) show the Hall resistance at 5 and 295 K for Sb_2_Te_3_ and Bi_2_Te_3_, respectively.

Figure 1 shows the temperature-dependence of the resistivity (ρ) for both the Sb_2_Te_3_ (Fig. 1a) and Bi_2_Te_3_ (Fig. 1b) films in the 5–295 K range.

As can be observed in Fig. 1, both the films show a metallic behaviour with an observed total 40% and 60% decrease in the resistivity from 295 to 5 K, for Sb_2_Te_3_ and Bi_2_Te_3_ respectively, being in accordance with previous reports^25,30–33^. The character of the carriers is extracted from the slope of the Hall-voltage (see insets in Fig. 1), and it is found to be p-type and n-type respectively in Sb_2_Te_3_ and Bi_2_Te_3_^30,33,34^. The carrier density (*d(n,p)*) and the mobility (*µ*) were also deduced from the Hall resistance as described by Eqs. (2) and (3) (see Methods) and the obtained values are reported in the Supplementary Information. From the metallic behaviour of both the films, it is evinced that the conduction from bulk states (BS) plays a fundamental role in the electronic transport mechanism, differently from the theoretical prediction^35^. Nevertheless, the fabrication of thin films where the bulk conduction is totally suppressed requires an extreme precision in the engineering of the Fermi level position, which constitutes a remarkable challenge. The main technical issue concerns the typical narrowness of the chalcogenide-based TIs band^1,27^ and the unintentional doping provided by the presence of defects or by slight stoichiometric variations. Due to these reasons, in absence of a fine band structure engineering, TIs often show a metallic character dominated by the BS^34,36–38^.

### Magnetoconductance characterization and WAL analysis

**Figure 2:**
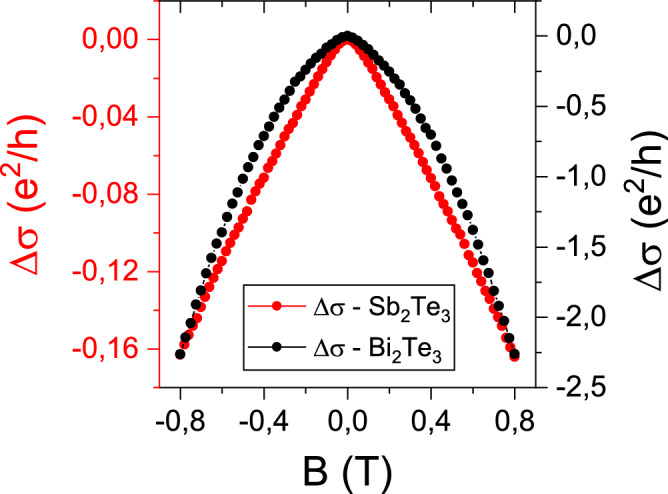
Comparison of the MC curves for the Sb_2_Te_3_ (red dots) and Bi_2_Te_3_ (black dots) films obtained at 5.5 K.

By observing Fig. 2, a remarkable difference in the absolute value of the MC amplitude in Sb_2_Te_3_ and Bi_2_Te_3_ could be noticed, with the latter showing a signal higher by more than one order of magnitude. Such a difference, jointly with the more-parabolic shape of the MC curve for the Bi_2_Te_3_ film, suggests that here there is a larger contribution to the overall transport from the BS when compared with the Sb_2_Te_3_ film.

In order to investigate the WAL, the parameters α and coherence length (*l*_Ф_) are extracted by applying the HLN model, as reported in Eq. (4), where α is connected to the number of conductive channels in which the transport mechanism is driven by quantum diffusion and the *l*_Ф_ represents the average distance along which the spin phase is conserved. Within the HLN model, a negative α value is associated to the presence of WAL, with α =  − 0.5 in the hypothesis that WAL originates from the TSS belonging to one of the two TI surfaces, or α =  − 1 if both contribute to the electronic transport^34^. Values of α different from the previous ones indicate a mixed situation, where the transport across the TSS is not ideal due to the intermixing between the BS and TSS. The C parameter expressed in Eq. (4) was then employed to remove the BS contribution from the measured MC, as clarified in the Methods, to highlight the signal resulting from the quantum correction related to the presence of WAL.

**Figure 3:**
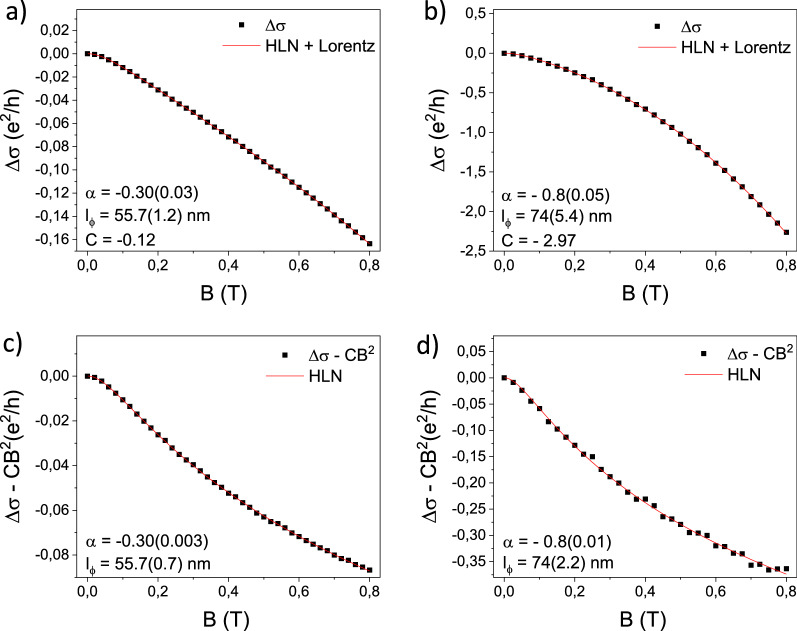
In panels (**a**) and (**b**), the MC curves measured at 5.5 K and fitted with the HLN equation comprehensive of the quadratic term are reported for Sb_2_Te_3_ and Bi_2_Te_3_ respectively. In (**c**) and (**d**) the MC signals, as shown in (**a**) and (**b**), are displayed removing the parabolic contribution to the fit for Sb_2_Te_3_ and Bi_2_Te_3_ respectively, and the HLN fit is performed again without the quadratic term.

In Fig. 3a,b, the acquired MC curves for the samples of Sb_2_Te_3_ and Bi_2_Te_3_ are reported, where the red solid line indicates the fit performed using Eq. (4) for each dataset, respectively. The same MC data upon the subtraction of the quadratic part as previously extracted (C parameter of Eq. 4) are displayed below in panels (c) and (d) of the same Fig. 3.

The |α| values extracted for the Sb_2_Te_3_ and Bi_2_Te_3_ films are 0.3 and 0.8 respectively, suggesting that the contribution of carriers driven by quantum diffusion is larger in Bi_2_Te_3_ than in Sb_2_Te_3_. Accordingly, *l*_Ф_ = 74 nm in Bi_2_Te_3_ and *l*_Ф_ = 55 nm in Sb_2_Te_3_. On the other hand, the higher C parameter extracted for Bi_2_Te_3_ indicates that the BS are relevant in the conduction mechanisms in such a film. Removing the quadratic term in Eq. (4), which approximate the contribution of the BS in the MC curve, the typical cusp-like MC is clearly observed in both materials, as reported for many TIs where the Fermi level is placed exactly in the centre of the BS band gap^1,13^. The results for α and *l*_Ф_, obtained from the signals cleaned from the BS contribution, are in perfect agreement with the results obtained on the rough data, validating the method.

To quantitatively discuss the difference in the shape of the MC curves, the parameter $\gamma$ is introduced and it is defined as the ratio between the MC with the quadratic contribution subtracted, and the measured MC, both evaluated at the maximum magnetic field (0.8 T).

1 $$\gamma = \frac{{\left( {\Delta\upsigma - CB^{2 } } \right)\left[ {0.8T} \right]}}{{\Delta\upsigma \left[ {0.8T} \right]}}$$

$\gamma$ is equal to 0.16 for Bi_2_Te_3_ and 0.5 for Sb_2_Te_3_. This further support that in Bi_2_Te_3_ the conduction from the BS is more relevant than in Sb_2_Te_3_ (i.e., giving a larger classical contribution) as revealed by ARPES (see "Angle-resolved photoemission spectroscopy (ARPES)").

Considering the HLN parameters extracted at 5–6 K for the MBE-grown Bi_2_Te_3_^30,39^, the α and *l*_Ф_ values obtained from our films turns out to be comparable. Differently, the α and *l*_Ф_ values detected in Sb_2_Te_3_ are lower than those previously reported for Sb_2_Te_3_ films grown by MBE^34^. The different HLN parameters obtained for Sb_2_Te_3_ and Bi_2_Te_3_ can be interpreted in terms of their crystalline quality, being the latter ordered in a more epitaxial crystalline structure. In fact, in our previous publications^18,19^, the crystal structure of MOCVD-grown Sb_2_Te_3_ and Bi_2_Te_3_ was carefully investigated. The XRD pattern was collected in the Bragg–Brentano geometry, as shown in Fig. 4 of Ref.^18^ and in Fig. 2 of Ref.^19^, where, in both cases, the Full Width at Half Maximum (FWHM) of the [006] peak was measured along the rocking angle axis. In this experimental configuration, the evaluation of the FWHM provides information about the mosaicity of the crystal, which measures the broadening of the out-of-plane orientation of the crystalline grains of a film. For rhombohedral crystals such as Sb_2_Te_3_ and Bi_2_Te_3_, a lower mosaicity implies a better alignment of the c-axis (out-of-plane) of the crystals lattice. The two reported values, 0.46° for Sb_2_Te_3_ and 0.26° for Bi_2_Te_3_, suggest that the latter is composed by crystals with a lower dispersion around the c-axis and thus more aligned to each other.

**Figure 4:**
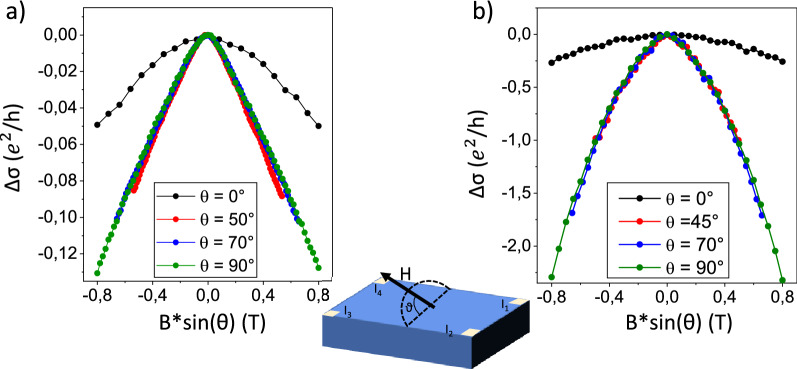
MC curves recorded at 5.5 K with the magnetic field applied at different angles with respect to the surface of Sb_2_Te_3_ in (**a**) and Bi_2_Te_3_ in (**b**). In both the panels the signal reported for ϑ = 0° is plotted as a function of the in-plane field since in that configuration there is not a perpendicular component.

To investigate the origin of the observed WAL, a dedicated experiment is performed to distinguish between 2D-originated WAL or 3D-originated WAL, as described in the methods section. Figure 4 reports the MC curves recorded with the field applied at different angles (θ) with respect to the plane of the samples and plotted as a function of the perpendicular component of the magnetic field (B sin(θ)). In the adopted reference frame, θ = 0° is obtained when the field is applied in the plane of the sample and θ = 90° when the field is perpendicular to the plane of the sample.

From Fig. 4, it can be noticed that, even at θ = 0°, a parabolic signal is still recorded, demonstrating that in such configuration it exhibits a circular motion superimposed to the electronic drift. This represents another indication that BS play a role in the transport mechanism of both the films and thus the magnetic Lorentzian force could induce a cyclotronic orbit even on planes not parallel to the surface of the TIs. On the other hand, being the presence of electrons in the TSS inherently 2D-confined (at the surfaces of the film and/or interfaces with the substrate), any field component at θ ≠ 90°, cannot excite any circular motion. In other words, the θ = 0° magnetic field component can only generate MC effects originating from the BS. The previous considerations indicate that the MC signals, which are proven to be directly proportional to the perpendicular component of the magnetic field, can be associated only to 2D conduction states^38^. For these reasons, the observed good overlapping between the signals, recorded over a wide range of angles of the magnetic field with respect the sample surface and plotted as a function of the perpendicular component of the magnetic field (Fig. 4), is a strong indication that the observed WAL originates from 2D conduction states.

Following the typical interpretation for the value of α, in the case of Sb_2_Te_3_, its absolute value lower than 0.5 indicates that just one interface of the film exhibit Dirac-like band structure. Therefore, a possible interpretation for the obtained value of |α|= 0.3 is that at 5.5 K, it exists an intermixing between the 2D Dirac-like band and the BS. Differently, for the Bi_2_Te_3_ films the obtained |α| value is very close to 1 and this could be associated with the presence of two interfaces showing conduction driven by 2D Dirac-like bands. The *l*_Ф_ is of the same order of magnitude in both the films, but it results slightly larger in Bi_2_Te_3_, meaning that here the electrons can conserve their spin state over a larger distance, accordingly to the lower BS-TSS intermixing already indicated by the different values of α. From the above reported experiments, it could be inferred that the WAL investigated is due to the existence of 2D transport channels whose conduction is driven by quantum mechanism. On the other hand, the direct visualization of the band structure is needed, as discussed in the next section. The HLN parameters were also extracted in the 5–25 K temperature range and the results are reported in the Supplementary Information.

### Angle-resolved photoemission spectroscopy (ARPES)

**Figure 5:**
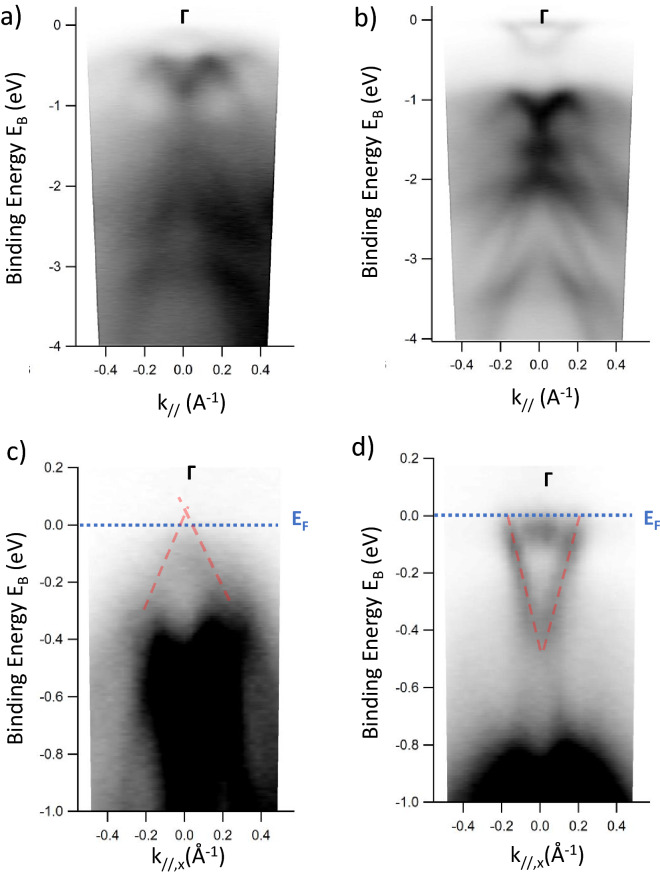
Angle-resolved photoemission spectroscopy intensity maps obtained at RT for Sb_2_Te_3_ in (**a**, **c**) and for Bi_2_Te_3_ in (**b**, **d**). Panels (**c**) and (**d**) show a detail of the band structure close to the Fermi level covering energy values from 0.2 to − 1 eV. The Fermi level is placed at zero energy and the connections between the valence and conduction band, i.e., the TSS, are highlighted by the red dashed line.

ARPES measurements have been conducted at room temperature (RT) on both Sb_2_Te_3_ and Bi_2_Te_3_, following appropriate preparation of the surface of the sample as detailed in the Supplementary Information. In Fig. 5, the electronic band structures of Sb_2_Te_3_ (panel (a,c)) and Bi_2_Te_3_ (panel (b,d)) around the Γ point of the Brillouin zone are imaged giving results which are in accordance with previous reports^1,27–29^.

In Fig. 5 (panels (c,d)), the surface states are highlighted with the red dashed lines. In the case of Sb_2_Te_3_ (Fig. 5c), the dirac point (DP), located at the crossing of the surface states, is very close to the Fermi level (less than 0.1 eV above it), thus, approaching the ideal situation in which the contribution of the TSS is dominant in the transport mechanism. This behaviour is also in agreement with the very marginal parabolic contribution observed in the corresponding MC curves (see Figs. 2, 4). In the case of Bi_2_Te_3_ (Fig. 5b,d), the intensity map clearly evidences that the Fermi level crosses the conduction band (~ 0.5 eV above the DP), and therefore the conduction is characterized by the superposition of TSS-related transport and BS-related transport. Considering that ARPES is known to be a surface-sensitive technique, the observation of such a Dirac-like band structure proves that the topological states are located at the surface of the films, and not buried between the quintuple layers composing the bulk.

Moreover, considering the intercept between the Fermi level (blue dashed line in Fig. 5c,d) and the TSS (red dashed lines in Fig. 5c,d) it is possible to estimate the Fermi wavevector k_F_. Within the semiclassical Drude model, k_F_ can be exploited to extract the electronic MFP, as detailed in the Supplementary Information. The estimated MFP at low temperature is ~ 4 nm for Sb_2_Te_3_ and ~ 75 nm for Bi_2_Te_3_. As reported in Ref.^25^ the WAL effect is more evident in materials where *l*_Ф_ > > MFP, being in accordance with the obtained MC results (see Figs. 2, 3), where the WAL effect is quite clear for the Sb_2_Te_3_ while barely visible in the Bi_2_Te_3_ film. This result is also in perfect agreement with the higher γ = 0.5 observed in Sb_2_Te_3_, when compared with the γ = 0.16 obtained for Bi_2_Te_3_ where the Fermi level crosses the conduction band thus enhancing the BS contribution in the transport mechanism.

**Figure 6:**
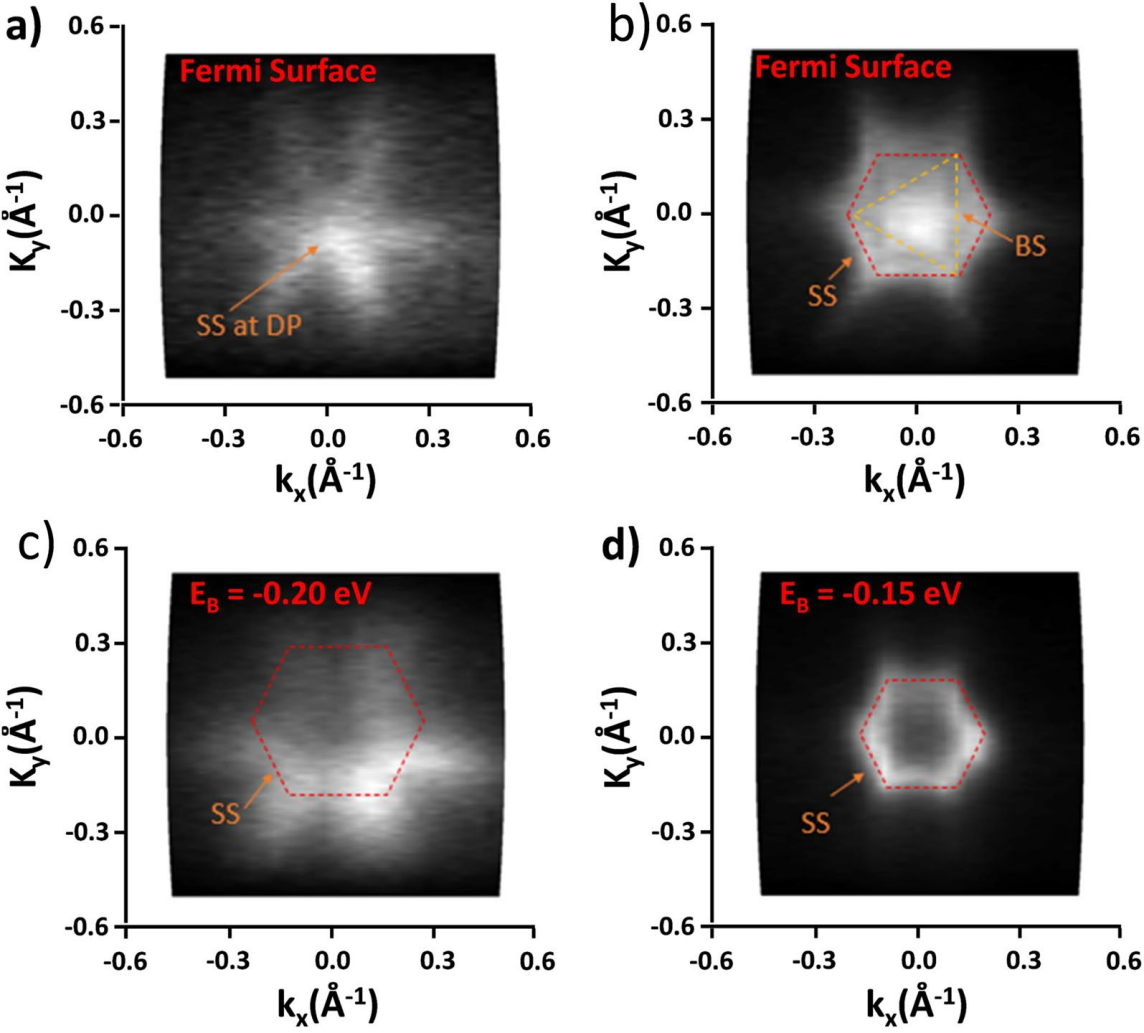
k_x_–k_y_ constant energy contour plots obtained at different constant binding energies. In (**a**) and (**c**) it is shown the result for Sb_2_Te_3_ at the Fermi level and 0.2 eV below it, respectively. In (**b**) and (**d**) it is shown the polar map of Bi_2_Te_3_ at the Fermi energy and 0.15 eV below it, respectively. The data shown in (**a**, **c**) are taken from Ref.^22^.

Figure 6 shows the *k*_*x*_–*k*_*y*_ constant energy contour plots (CECPs) for Sb_2_Te_3_ (panels (a,c)) and Bi_2_Te_3_ (panels (b,d)), as recorded at the Fermi level (panels (a,b)) and below it (panels c,d).

From Fig. 6a, it can be noticed that the spectrum shows a high-intensity point associated with the connection between the two cones (i.e., the DP) along with a hexagonal symmetry feature, which is compatible with a 2D-TSS obeying the time-reversal symmetry. Decreasing the energy by 0.2 eV (see Fig. 6c), the high intensity point is no more visible, and the spectrum preserves its hexagonal symmetry, indicating that the BS are not yet crossed. On the other hand, for the Bi_2_Te_3_ case, the spectrum obtained at the Fermi energy (see Fig. 6b) shows a feature with a trigonal symmetry (orange dashed line) reflecting the trigonal symmetry of rhombohedral bulk Bi_2_Te_3_ (BS signature), surrounded by a faded hexagonal pattern that originates from the TSS. At a decreased energy of −0.15 eV with respect to the Fermi level (see Fig. 6d), the bulk trigonal symmetry is no more visible, with the clear hexagonal symmetry associated with the TSS now dominating and showing a smaller hexagon as a further demonstration that the constant energy contour is now closer to the DP.

## Conclusion

By combining temperature-dependent (5–295 K) magnetotransport and room-temperature ARPES, the topological character of large-area Sb_2_Te_3_ and Bi_2_Te_3_ thin films grown by MOCVD on 4″ Si(111) substrates, has been validated. MC curves display WAL effects in both materials, with a larger contribution from the BS observed in Bi_2_Te_3_. By interpreting the MC data within the Hikami–Larkin–Nagaoka model, the parameters α and *l*_Ф_ are extracted at 5.5 K resulting in an α equal to 0.3 and 0.8 for Sb_2_Te_3_ and Bi_2_Te_3_ respectively and a coherence length *l*_Ф_ of 55 nm and 75 nm for Sb_2_Te_3_ and Bi_2_Te_3_, respectively. Angular-dependent MC experiments clearly confirm that the observed WAL effect originates uniquely from the 2D states in both Sb_2_Te_3_ and Bi_2_Te_3_, thus making possible to attribute the extracted α and *l*_Ф_ parameters to the 2D Dirac-like bands. Approximate energy gaps between the DP and the Fermi level were estimated from the ARPES spectra, resulting in less than 0.1 eV in the Sb_2_Te_3_ and around 0.5 eV in the Bi_2_Te_3_. In the latter, the Fermi level completely crosses the bulk conduction states, and this is well in accordance with the extracted γ values (0.5 in Sb_2_Te_3_ and 0.16 in Bi_2_Te_3_) remarking the presence of a more relevant bulk contribution to the transport in Bi_2_Te_3_.

We unambiguously demonstrate that the Sb_2_Te_3_ and Bi_2_Te_3_ thin films grown by MOCVD on large areas (up to 4") Si(111) substrates are topological insulators. Our results are important in view of achieving their realistic technology transfer, being the MOCVD method already widely employed in semiconductor industry.

## Methods

The Sb_2_Te_3_ and Bi_2_Te_3_ thin films with epitaxial quality are grown by MOCVD at RT and 350 °C, respectively^18,19^. Both layers are grown on Si(111) substrates previously treated with hydrofluoric acid (HF 5% in deionized water) to remove surface contaminants. Moreover, only for the Sb_2_Te_3_, to guarantee the total desorption of any organic molecule from the Si surface, prior to the film deposition an additional annealing was conducted at 500 °C, being the growth performed at RT. The deposition process adopted to grow the studied samples has been already optimized with the aim of producing the films as thin as possible still guaranteeing their overall morphological continuity and chemical-structural quality (i.e., epitaxial fashion)^18,19^. A detailed crystallographic analysis is reported in Refs.^18,19^, where X-ray diffraction revealed the highly oriented rhombohedral crystalline structure of the studied materials, belonging to the $R\overline{3 }m$ space group (i.e., ICSD 74348 and 2084)^18,19^. Here, the thickness of the samples was measured by X-ray reflectivity for Sb_2_Te_3_ (Figure S23 and Table S1 of the SI of Ref.^18^) being equal to ~ 32 nm, and by scanning electron microscopy cross section images for Bi_2_Te_3_ (Fig. 1 of Ref.^19^), being equal to ~ 90 nm. TEM and XRR analysis were also employed to show the presence of an atomically sharp and chemically pure interface, therefore excluding both any major chemical mixing at the Si(111)/TI interface and the presence of unwanted inorganic contaminants in the material. Additionally, atomic force microscopy (AFM) was conducted on a 0.5 × 0.5 μm^2^ area in both samples, and a surface roughness of ~ 1 nm was found for Sb_2_Te_3_ and ~ 0.5 nm for Bi_2_Te_3_^18,19^. All the reported investigations were carried out on macroscopic samples obtained by cutting the 4’’ wafer on which the TIs are deposited. Prior to the methodic characterization performed on a single sample, for each MOCVD run different parts of the growth area were investigated to check their crystallographic and morphological quality, ensuring the reproducibility of the obtained results.

Magnetotransport measurements were performed in the Van der Pauw configuration on ~ 1 × 1 cm^2^ samples in the 5–295 K temperature range, without any prior processing or capping layers. The latter measurements were conducted at a constant applied current as provided by a Keitlhey 2610, with the voltage recorded by a Keitlhey 2182A nanovoltmeter. External magnetic field up to 0.8 T was applied at a variable direction with respect to the samples plane. In particular, the magnetic field has been applied both in parallel (0°) and perpendicular (90°) direction with respect to the surface of the sample to investigate the conduction mechanism.

The measured ordinary resistivity (ρ) has been combined with the Hall effect signal, to calculate the mobility ($\mu$) and the density of the electrons or holes carriers ($d\left( {n,p} \right)$) as indicated in Eqs. (2) and (3), respectively.

2 $$d\left( {n,p} \right) = \frac{IB}{{teV_{H} }}$$

3 $$\mu = \frac{1}{{ed\left( {n,p} \right)R_{s} }}$$

where $V_{H}$ is the Hall voltage, *e* is the electron charge (*e* = 1.6 × 10^−19^ C), *t* is the thickness of the film, $R_{s}$ is the sheet resistance which is equal to *ρ* divided by the thickness of the film and B indicates the magnitude of the applied magnetic field. The nature of the carriers (*n* or *p*) was deduced by evaluating the slope of the recorded Hall voltage in a $V_{H}$(B) graph (see Fig. 1).

Further, the sheet resistance in the Van der Pauw configuration was analyzed as a function of the perpendicularly applied magnetic field. The measured resistance is plotted as a function of the magnetic field, and it is inverted to obtain the MC. To make the various measurement comparable to each other, it was defined the Δσ as the MC signal cleaned by a baseline acquired at zero magnetic field and subsequently expressed in unit of e^2^/h.

The MC signal is carefully analysed to investigate the presence of WAL, in the framework of the HLN model^24^, as a proof of the fact that, partially, the electrical transport is driven by quantum mechanism^25,26,34^.

The WAL effects constitute in a quantum correction of the MC data. In absence of such quantum correction the phenomena that define the MC shape are various. Superimposed to the classical contribution to the MC, elastic scattering events, the presence of impurities and the effect of SOI are taken into account by means of a Taylor expansion in the magnetic field truncated at the second order (C*B^2^)^40^. The typical WAL manifestation consists in the cusp-like dispersion MC, as reported for several materials characterized by strong spin-momentum correlation and high mobility^36,41^, as described by Eq. (4).

4 $$\Delta \sigma = - \alpha \frac{{e^{2} }}{\pi h}\left( {\Psi \left( {\frac{1}{2} + \frac{h}{{8\pi el_{\upphi }^{2} B}}} \right) - ln\left( {\frac{h}{{8\pi el_{\upphi }^{2} B}}} \right)} \right) + {\text{C}}^{*} {\text{B}}^{2}$$

where *Ψ* represents the digamma function, *h* is the Plank constant, *e* is the electron charge, *C* is the quadratic coefficient, *B* is the magnetic field and α and *l*_Ф_ are the HLN parameters. Being aware that the samples may present a different electrical behavior due to their different thicknesses, the WAL investigation was conducted by employing various experimental configurations to decouple the contribution arising from the 2D (i.e., surfaces) and 3D (i.e., bulk) quantum effects.

In fact, to reveal the origin of the observed WAL a dedicated experiment is performed exploiting the application of a magnetic field at various angles with respect to the plane of the sample. In this configuration, if the WAL effect is generated by 2D conduction channels, due to geometrical considerations, the MC measured must be proportional solely to the perpendicular component of the applied magnetic field^38^. In the present case, the thicknesses of the films are 30 and 90 nm for Sb_2_Te_3_ and Bi_2_Te_3_ respectively, thus the component of the MC arising from BS it is expected to show up also when the field is not perpendicular to the surface of the film. This is derived from the observation that the cyclotronic orbit, directly related to the existence of the Lorentzian force ($\propto \vec{v} \times \vec{B}$), is responsible for the deviation of part of the charge from their ordinary drift direction. Thus, inducing a BS-related magnetoresistive effect observable with field applied parallel to the plane of the sample^38^.

Following the magnetotransport characterization, the same samples were analysed by *ex-situ* ARPES. Due to the partial oxidation of the Sb_2_Te_3_ and Bi_2_Te_3_ surfaces, the samples were cleaned under vacuum condition by 1.5 keV Ar ion sputtering at 10^5^ mbar prior to ARPES measurements. The sputtering duration was settled to obtain a clean surface, free of O contaminants, as verified by in-situ X-ray photoemission spectroscopy (see Supplementary Information). As a final step, an annealing process under vacuum was performed with the aim to recover the damage induced by Ar^+^ sputtering. Finally, flat and well-ordered surfaces were obtained, as checked by streaky reflection high-energy electron diffraction patterns. ARPES spectra were acquired at RT with a 100 mm hemispherical electron analyzer equipped with a 2D CCD detector (SPECS). The He I (21.22 eV) resonant line was used to excite photoelectrons and the energy resolution of the system was larger than 40 meV. The spot of the employed ARPES facility has an elliptical shape with an area of about 4 × 6 mm^2^. As a consequence, the ARPES characterization conducted on our samples makes possible to extract information about the dispersion of the materials band structure on a relatively macroscopic area, thus guaranteeing a certain uniformity of the topological nature of the conductive state over the investigated TI’s area.

## Supplementary Information

Supplementary Information.
