# Supplementary material for: Magnetotransport and ARPES studies of the topological insulators Sb2Te3 and Bi2Te3 grown by MOCVD on large-area Si substrates
Source: Sci Rep. 2022 Mar 10;12:3891. doi: 10.1038/s41598-022-07496-7 (PMC8913753; doi:10.1038/s41598-022-07496-7)
Supplement: Supplementary file 1 — Supplementary Information. [file 41598_2022_7496_MOESM1_ESM.docx]

Supplementary Information

Magnetotransport and ARPES studies of the topological insulators Sb_2_Te_3_ and Bi_2_Te_3_ grown by MOCVD on large-area Si substrates

*L. Locatelli^1,2*^, A. Kumar^1^, P. Tsipas^3^, A. Dimoulas^3^, E. Longo^1^, and R. Mantovan^1*^*

^1^ Institute for Microelectronics and Microsystems, CNR-IMM Unit of Agrate Brianza, Via C. Olivetti 2, 20864, Agrate Brianza, Italy

^2^ University of Milano Bicocca, Department of material science, Via R. Cozzi 55,20126, Milano, Italy

^3^ National Centre for Scientific Research 'DEMOKRITOS', Patriarchou Grigoriou & Neapoleos 27, 15310, Agia Paraskevi, Athens, Greece

*E-mail: [lorenzo.locatelli@mdm.imm.cnr.it](mailto:lorenzo.locatelli@mdm.imm.cnr.it), and [roberto.mantovan@mdm.imm.cnr.it](mailto:roberto.mantovan@mdm.imm.cnr.it)

**Temperature dependent mobility and carrier density**

The carrier density d(n,p), the µ and the MFP are tracked as a function of the temperature and shown in Figure S1 for both Sb_2_Te_3_ (a,c) and Bi_2_Te_3_ (b,d).


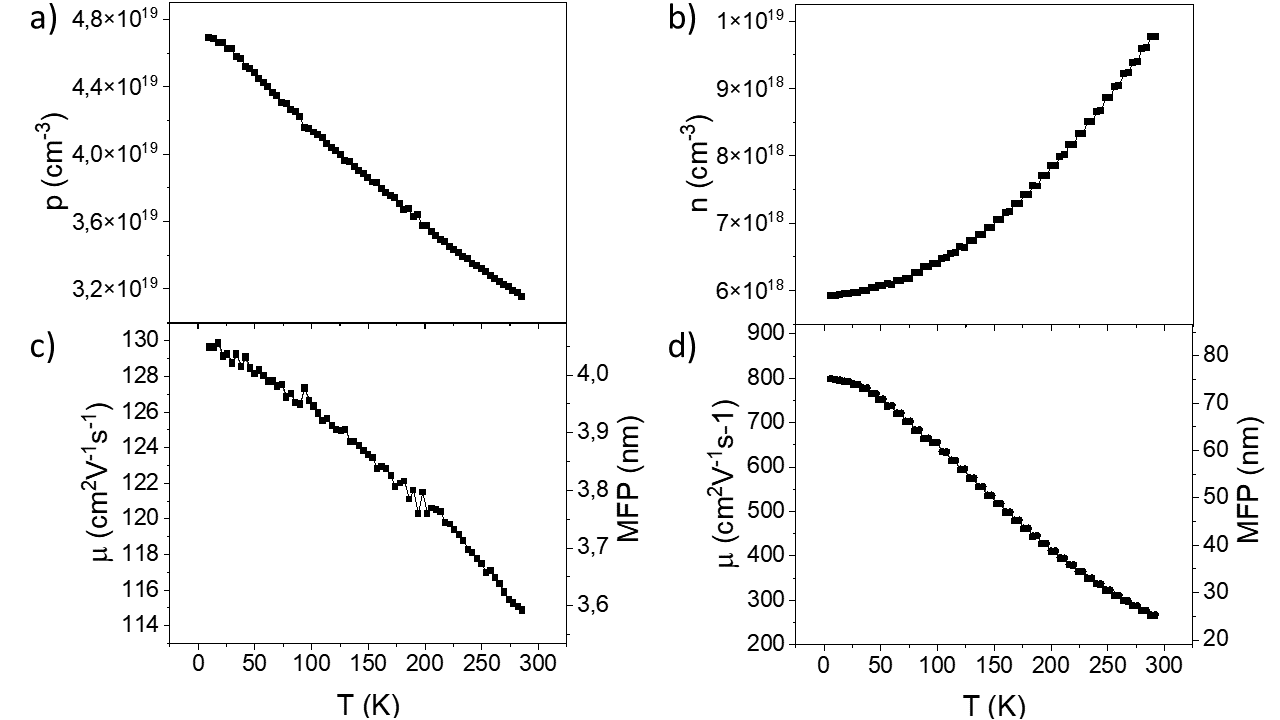


**Figure S1**: Electrical parameters evolution in the 5 – 295 K range: In panel (a) the hole density, in panel (c) the µ (left scale) and the MFP (right scale) for Sb_2_Te_3_. In panel (b) the electron density for Bi_2_Te_3_, in panel (d) the µ (left scale) and the MFP (right scale) for Bi_2_Te_3_.

The order of magnitude of the carrier densities is 10^19^ cm^-3^, which is in accordance with the 3D carriers’ concentrations reported for many TIs, and usually associated with the presence of conductive defects in the BS and unintentional doping^1,2^. Indeed, a typical value for densities of carriers associated to 2D conduction channels should be $n_{2D}=1\times{10}^{11-12}{cm}^{-2}$, and it is usually extracted by measuring the frequency of the Shubnicov de Haas oscillations^3,4^ which cannot be observed at the relatively low magnetic field values allowed in our experimental setup.

Sb_2_Te_3_ exhibits lower µ in comparison to the Bi_2_Te_3_ films over the whole range of temperature accordingly to the better overall crystallographic quality, as discussed in the main text. Focusing on the value registered at low temperature, in the case of Bi_2_Te_3_, there are reported values that cover a large range spanning from 500 cm^2^/Vs ^5^ to 4 $\times$10^4^ cm^2^/Vs ^6^. In the case of Sb_2_Te_3_ the reported values in the literature are found around 500 cm^2^/Vs ^7,8^. Our values for both the materials are generally lower than the reported for MBE-grown TIs, again indicating that such parameter is strongly affected by the crystallographic quality.

Aiming to provide an estimation of the MFP for both the materials, we make use of equation S1 as derived from the semiclassical Drude model:

$$MFP= \frac{\mu k_{F}ћ}{q} S1$$

Where k_F_ is the Fermi wavevector, $ћ$ is the reduced Plank constant and q is the charge. The k_F_ can be extracted from Figure 5(c,d) for Sb_2_Te_3_ and Bi_2_Te_3_ respectively as the crossing point between the TSS (red dashed lines) and the Fermi level being ~ 0.05 Å^-1^ for Sb_2_Te_3_ and ~ 0.15 Å^-1^ for Bi_2_Te_3_. The µ value for both the films is extracted from the Hall measurement and reported as a function of the temperature in Figure S1(c) for Sb_2_Te_3_ (left scale) and S1(d) for Bi_2_Te_3_ (left scale). Considering that the ARPES spectra are taken at RT, in order to derive the MFP in the 5 – 290 K temperature range, we assume constant k_F_ as a function of the temperature, with only the bands occupation state varying. The derived MFPs are depicted in Figure S1.

**Temperature dependent HLN parameters**

In order to investigate on the evolution of the degree of intermixing between the TSS and BS in both the Sb_2_Te_3_ and Bi_2_Te_3_ films, in Figure S2, the evolution of the α and *l*_Ф_ parameters as a function of the temperature is reported, as extracted from the HLN model using Equation 4. From Figure S2(a), the Sb_2_Te_3_ displays a sligth reduction of the α parameter when the temperature increases from 5.5 to 25 K. This behavior suggests that with the increasing of the temperature the BS interfer more efficiently in the electronic conduction, affecting the pure 2D Dirac-like transport in Sb_2_Te_3_^9,10^. On the other hand, Figure S2(b) evidences how in the Bi_2_Te_3_ films the α slightly increases until saturation in the 5-25 K temperature range, demontrating a more stable topological character in such a film, as also reported by others^11^. The evolution of the *l*_Ф_ parameter as a fuction of the temperature is reported in Figures S2(c) and (d) for Sb_2_Te_3_ and Bi_2_Te_3_ respectively. Here, a general decreasing of *l*_Ф_ is observed as a function of the increasing temperature for both the studied films, as expected^12^. In particular, for ideal condition of completely indipendent TSS and BS, the *l*_Ф_ should be proportional to T^-p^ with p=0.5 as predicted by the Nyquist dephasing rate due to the 2D electron-electron interaction^9,11,13,14^. In our case, the determination of the *p*  parameter is not conclusive, due to the botton limit of the temperature range explored.


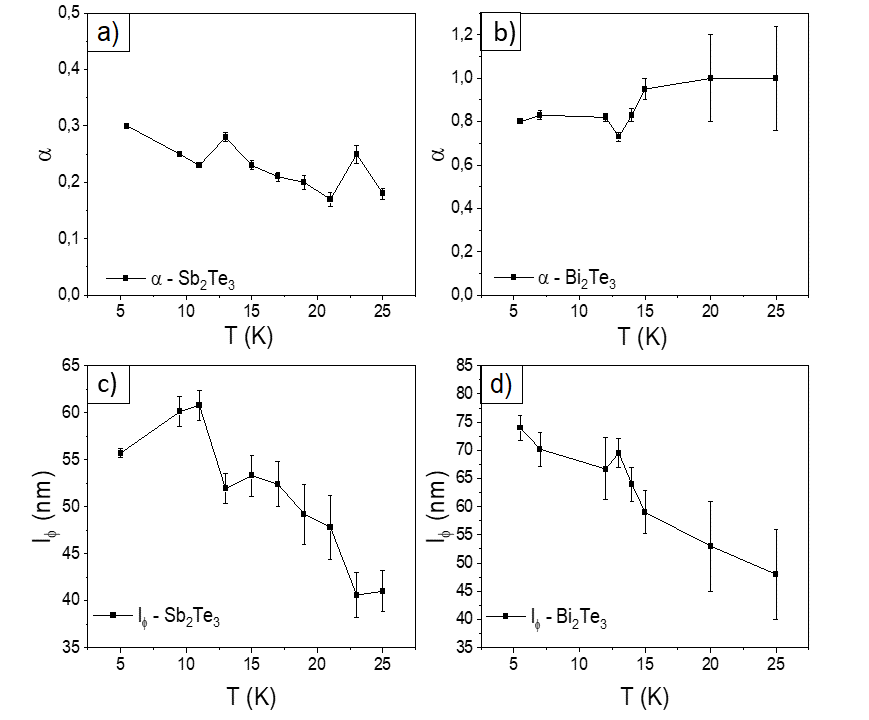


**Figure S2**: α (a,b) and l_Ф_ (c,d) for Sb_2_Te_3_ and Bi_2_Te_3_ respectively, the parameters are extrapolated by fitting the Δσ with the equation 2, for some relevant temperatures.

The obtained results indicate that the coupling between BS and TSS is quite stable as a function of the temperature in both the films. This stability is fundamental to predict that such TSS could be detected also at room temperature, as it was recently confirmed by our work on spin-to-charge-conversion experiments performed at room temperature^15,16^. The upper temperature limit beyond which the investigation of WAL effect has been conducted is mainly related to the increasing of the error bars affecting the data at temperature above 25 K, particularly in the case of Bi_2_Te_3_. A precise identification of a temperature at which the WAL effect disappears is not straightforward for the studied TIs, since both the films present a MC signal composed by a clear sum of a quadratic contribution arising from the BS and a non-parabolic part due to the TSS. For instance, in the case of the Sb_2_Te_3_ the parabolic contribution is less relevant than in Bi_2_Te_3_, thus the evolution of the MC signal as a function of the temperature is evident. As an example, in Figure S3(a) the signal at 38 K displays a clearly parabolic shape, suggesting that at this temperature the TSS contribution is no more magnetoelectrically observable. On the other hand, being the MC signal arising from the Bi_2_Te_3_ heavily affected by the parabolic BS contribution also at low temperatures, such a difference is less evident from a qualitative comparison of the shape of the MC signals at 5.5 K and 40 K (see figure S3(b)).

*
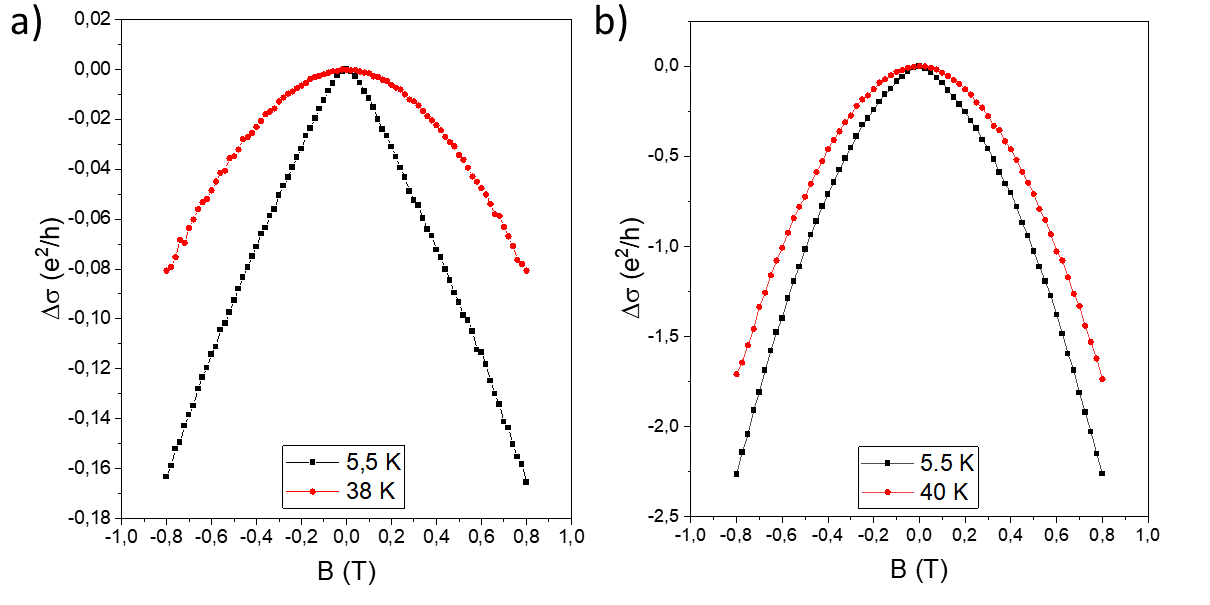
*
**Figure S3**: MC shown at different temperatures for Sb_2_Te_3_ in (a) and Bi_2_Te_3_ in (b).

***Ex-situ* ARPES measurements**

To conduct Angle Resolved Photoemission Spectroscopy (ARPES) was quite a challenge, since it was performed *ex situ* following the MOCVD growth, for both the Sb_2_Te_3_ and Bi_2_Te_3_ samples. In particular, no capping layers has been employed, and, consequently, the layers were partly oxidized when moved to the ARPES chamber.

To remove the surface contaminants, we sputtered the surface of Sb_2_Te_3_ with 1.5 KeV Argon ion at 10^5^ mbar and then annealed in vacuum at 292 °C. By means of X-ray photoemission spectroscopy (XPS), we tracked the surface’s modification following various sputtering duration and annealing. Figure S4 shows the change of the binding energy of the 3d orbitals for (a) Sb and (b) Te.


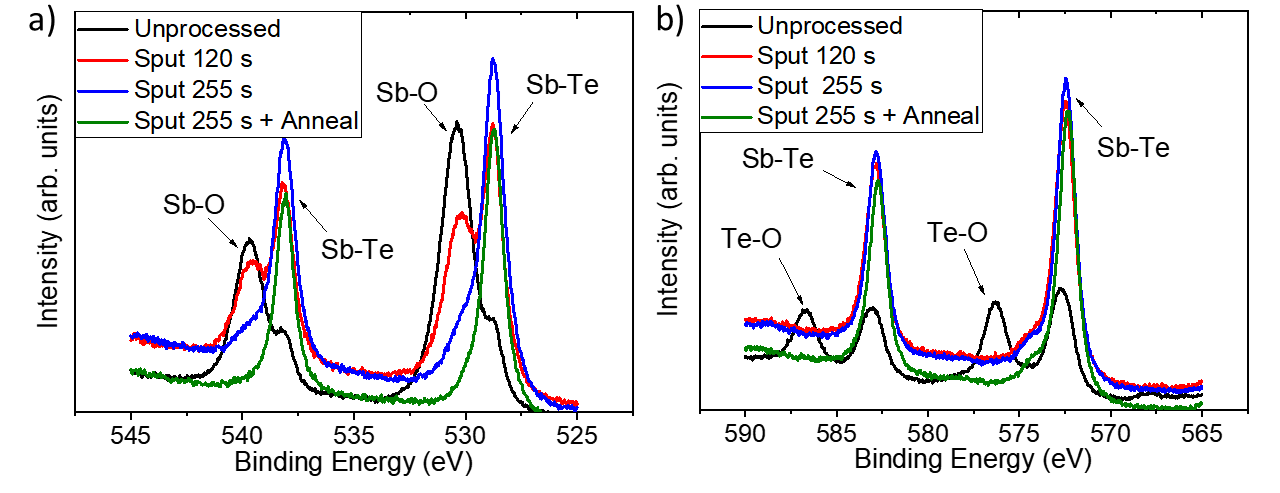


**Figure S4**: XPS spectra obtained on Sb_2_Te_3_ probing the 3d orbitals of (a) Sb and (b) Te. The various spectra are taken after several sputtering process (as depicted in the legenda) until the energy of the bonds Te-O and Sb-O vanishes.

From Figure S4, it can be noticed that the intensity peak related to the oxidation of both Sb and Te, is totally suppressed following the sputtering time of 255 s and a subsequent annealing, a process that turned out successful to fully recover the original Sb_2_Te_3_ surface. With this condition achieved, ARPES has been conducted.

By following the same procedure employed for Sb_2_Te_3_, also the surface of Bi_2_Te_3_ surface was reconstructed by an Ar sputtering followed by thermal annealing in vacuum at 292 °C, as depicted in Figure S5.


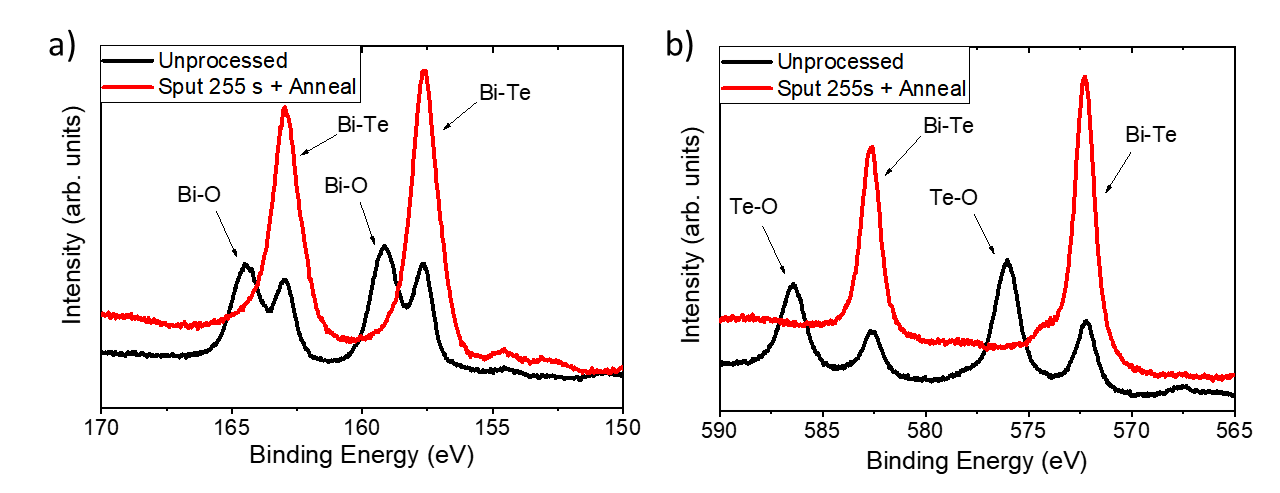


**Figure S5**: XPS spectra obtained on the Bi_2_Te_3_ film by probing (a) the 4f orbitals of Bi and (b) the 3d orbital of Te. The duration of the sputtering was already optimized here so it is shown just the difference between spectra of the unprocessed sample covered with oxide and the cleaned surface.

In a complete analogy to what is observed at the surface of Sb_2_Te_3_ (Fig. S4), also the Bi_2_Te_3_ surface is fully recovered following the adopted recipe, and ARPES has been successfully performed once the layers conditions depicted in Fig.S5 have been achieved.

**References**

1. Bendt, G., Zastrow, S., Nielsch, K., … P. M.-J. of M. & 2014, U. Deposition of topological insulator Sb 2 Te 3 films by an MOCVD process. *Journal of Materials Chemistry A* **2**, 8215–8222 (2014).

2. Zastrow, S. *et al.* Thermoelectric transport and Hall measurements of low defect Sb2Te3 thin films grown by atomic layer deposition. *Semiconductor Science and Technology* **28**, (2013).

3. Hamdou, B., Gooth, J., Dorn, A., Pippel, E. & Nielsch, K. Surface state dominated transport in topological insulator Bi2Te3 nanowires. *Applied Physics Letters* **103**, 193107 (2013).

4. Ngabonziza, P., Wang, Y. & Brinkman, A. Bulk contribution to magnetotransport properties of low-defect-density Bi2Te3 topological insulator thin films. *Physical Review Materials* **2**, (2018).

5. He, H. T. *et al.* Impurity effect on weak antilocalization in the topological insulator Bi2Te3. *Physical Review Letters* **106**, 166805 (2011).

6. Shrestha, K. *et al.* Extremely large nonsaturating magnetoresistance and ultrahigh mobility due to topological surface states in the metallic Bi2Te3 topological insulator. *Physical Review B* **95**, (2017).

7. Kampmeier, J. *et al.* Selective area growth of Bi2Te3 and Sb2Te3 topological insulator thin films. *Journal of Crystal Growth* **443**, 38–42 (2016).

8. Takagaki, Y., Giussani, A., Perumal, K., Calarco, R. & Friedland, K. J. Robust topological surface states in Sb 2Te 3 layers as seen from the weak antilocalization effect. *Physical Review B - Condensed Matter and Materials Physics* **86**, 125137 (2012).

9. Chiu, S. P. & Lin, J. J. Weak antilocalization in topological insulator Bi2Te3 microflakes. *Physical Review B - Condensed Matter and Materials Physics* **87**, (2013).

10. Steinberg, H., Laloë, J.-B., Fatemi, V., Moodera, J. S. & Jarillo-Herrero, P. Electrically tunable surface-to-bulk coherent coupling in topological insulator thin films. *PHYSICAL REVIEW B* **84**, 233101–233102 (2011).

11. Roy, A. *et al.* Two-dimensional weak anti-localization in Bi2Te3 thin film grown on Si(111)-(7×7) surface by molecular beam epitaxy. *Citation: Applied Physics Letters* **102**, 163118 (2013).

12. Gracia-Abad, R. *et al.* Omnipresence of Weak Antilocalization (WAL) in Bi 2 Se 3 Thin Films: A Review on Its Origin. *Nanomaterials* **11**, 1077 (2021).

13. Altshuler, B. L., Aronov, A. G. & Khmelnitsky, D. E. Effects of electron-electron collisions with small energy transfers on quantum localisation. *Journal of Physics C: Solid State Physics* **15**, 7367 (1982).

14. Plucinski, L. *et al.* Robust surface electronic properties of topological insulators: Bi 2 Te3 films grown by molecular beam epitaxy. *Applied Physics Letters* **98**, (2011).

15. Longo, E. *et al.* Large Spin-to-Charge Conversion at Room Temperature in Extended Epitaxial Sb2Te3 Topological Insulator Chemically Grown on Silicon. *Advanced Functional Materials* 2109361 (2021) doi:10.1002/ADFM.202109361.

16. Longo, E. *et al.* Spin-Charge Conversion in Fe/Au/Sb2Te3 Heterostructures as Probed By Spin Pumping Ferromagnetic Resonance. *Advanced Materials Interfaces* 2101244 (2021) doi:10.1002/ADMI.202101244.
